# Chemo cured the cancer: what about the patient?

**DOI:** 10.1186/1470-7330-15-S1-O35

**Published:** 2015-10-02

**Authors:** Nina Tunariu

**Affiliations:** 1 Radiology Department, Royal Marsden Hospital, London, UK

## 

In 2012, two million cancer survivors were reported in the United Kingdom. Using a model of prevalence as a function of incidence, survival and population demographics, Maddams et al projected that by 2040, the number of cancer survivors in the United Kingdom will increase by approximately one million per decade[1]. Late effects of cancer treatment can come from chemotherapy, radiation and surgery. With the increasing number of people that undergo curative treatments or long times of cancer remission, and subsequent prolonged survival time, late effects of cancer treatment can become clinically evident decades after completion of therapy. Modern oncological treatment regimens often incorporate multiple agents whose deleterious effects might be additive or synergistic, making their identification and treatment more challenging.

The spectrum of late anti-cancer treatment effects is broad and includes vision and hearing loss, early menopause, cardiomiopathy, infertility, nephropathy, hepatitis and pseudocirrhosis, pneumonitis, neuropathy, cognitive impairment, osteoporosis, sexual dysfunction and increased risk of second malignancy. Childhood cancer survivors may also experience memory problems and learning disabilities and short stature, caused by arrested or impaired growth and vasculopathy.

A greater understanding of sometimes multifactorial mechanisms of injury can prolong the lives and improve life quality of those cured of their malignancy, but left with potentially devastating sequelae. A such example is the cardio-vascular toxicity experienced by patients secondary to the malignant process itself or its treatment[2]. Oncological treatments have been associated with life-threatening arrhythmia, ischaemia, infarction, and damage to cardiac valves, the conduction system, or the pericardium. The increased awareness of cardiac late toxicity has resulted in development of strategies[3] to monitor and to prevent or to mitigate the effects of cardiovascular damage[4].

Whilst the focus of oncological care continues to be on cure, there is a growing need of recognition that for many patients, cancer has become a chronic disease; the paradigm must shift from illness to optimum wellness. Management of long-term chemotherapy-related toxicity involves screening for symptoms, use of supportive medication, and referral for specialty consultation as needed. Survivorship experts have promoted risk stratification to determine the intensity and setting for post-treatment follow-up[5]. Thus prediction of both the life-threatening effects and the psychosocial morbidity and sexual dysfunction that can significantly impair quality of life represents a critical next step[6] to allow implementation of optimal long term programs and resources in cancer survivors care.
